# The Institutional Worker

**Published:** 1913-03-08

**Authors:** 


THE
### INSTITUTIONAL WORKER
**Being a Special Supplement to "The Hospital"**

### A Good Menu at 5s. 2d. per week.

THIS is a special hospital for women—beds 53, average number occupied during December 1912, 49; staff 34. *Sundays.*—Cold beef or mutton, potatoes, stewed prunes or figs, and milk puddings (in summer, or when fruit is cheaper, always fresh stewed fruit once a week). *Mondays.*—Stew, with carrots and turnips in, potatoes, and milk puddings. *Tuesdays.*—Roast beef, cabbage, potatoes, milk puddings. *Wednesdays.*—Cottage pies, potatoes, and milk puddings. *Thursdays.* — Stew, with vegetables, potatoes, milk puddings. *Fridays.*—Boiled or fried fish (cod), parsley or egg sauce, potatoes, and milk puddings. *Saturdays.*— Roast or boiled mutton, onion or caper sauce, potatoes, milk puddings. Breakfast, lunch, tea, and supper.—Tea or coffee, milk and sugar, and bread and butter is provided. Convalescent patients on ordinary diet are allowed milk, etc., besides milk puddings daily and 1 oz. of butter, bread *ad lib.* Patients may have brought in to them eggs, fruit, and tea cakes. The cost of food only per patient is 5s. 2d., including fuel, gas, light, and water, 7s. 4d. per week. The food is changed each week, so that the patients do not know what food is coming each day.—DONOVAN.

### Greengrocer's Bill.

THE following shows the amount of potatoes and other vegetables used during December in our institution :—

| | £ | s. | d. |
|---|---|---|---|
| Potatoes, 2½ cwt. at 11/- cwt. ... | 1 | 7 | 6 |
| Swede Turnips, 1½ cwt. at 1/- cwt. | 0 | 1 | 6 |
| Onions, 4½ stones at 1/- stone ... | 0 | 4 | 6 |
| Tomatoes, 4 boxes at 3/3 stone... | 0 | 13 | 0 |
| Sprouts, 7 lb. wkly., 28 lb. at -/2 lb. | 0 | 4 | 8 |
| Carrots, 14 lb. weekly, at -/1 lb. | 0 | 1 | 2 |
| Parsnips, 14 lb. weekly, at -/1 lb. | 0 | 1 | 2 |
| Savoys, 16 lb. weekly, at -/2 each | 0 | 2 | 8 |
| Beetroot, 12 lb. weekly, at -/1 lb. | 0 | 1 | 0 |
| Celery, 16 heads at -/2 each ... | 0 | 2 | 8 |

None of this produce was grown on our own land. Our number of beds is 32, but our numbers for December were about 16, including scarlet fever, typhoid fever, and diphtheria. Our staff numbers 14. — NORTH-COUNTRY FEVER.

### Distressed Lodging-house Keeper.

WE recommend you not to break up the home of the poor lady who has got into difficulties through ill-health. Her furniture and connection are her only assets, and instead of dealing out to her the money you have collected week by week, we feel sure you would help her more efficiently by having her rooms done up and advertising them to let, "without attendance," at a cheap rate which will give her a margin to live on. If we hear of anyone wanting rooms in a romantic part of the country for week-ends we will bear her in mind.—BALLADER.

### OUR BUREAU OF INFORMATION.

### Rules for Correspondents.

1. Every letter, on whatever subject, must be accompanied by the coupon to be cut from the back cover (inside page) of THE HOSPITAL, current issue, and must contain the name and address of the correspondent with pseudonym for publication if desired.
2. Replies by post cannot be given, save under exceptional circumstances at the Editor's discretion.
3. Letters from Approved Homes in reply to special needs published in the Bureau should state terms and full particulars, and be sent prepaid under cover to the Editor of the Bureau with name written across coupon for identification.
4. Proprietors of Homes which have not yet been entered on the List of Approved Homes, but have spare accommodation likely to suit special needs, are invited to write for an application form for registration. The fee for registration, which includes two announcements of the Home in the Bureau and other privileges, is 5s.
5. All communications to be addressed to the Editor of THE HOSPITAL, 28 Southampton Street, Strand, London, W.C., and marked "Bureau of Information."

### Invitation to Readers.

**The Editor is prepared to forward to applicants, without fee on either side, offers of accommodation from Homes on the Approved List suitable for medical and surgical cases, for the aged, for chronic or mental patients, for electrical treatment and massage, for convalescents and for children, at varying terms, in any required locality. Reduced terms can often be arranged. The special needs of any who may be sick or in difficulties are also made known in these columns free of charge.**

### Approved Homes Recently Added to List.

NOTE.—*No Home is added to the List without medical and other testimony to its good management.*

**Scotland.**—Albertville Nursing Home, Musselburgh. Principal: Mrs. Stirling. Medical, surgical, chronic, and convalescent cases. Good accommodation for elderly ladies or gentlemen. Garden; sea air; near good medical baths.

**Sussex.**—57 Marina, St. Leonards-on-Sea. Principal: Miss South. Fully-equipped Medical and Surgical Home. Dowsing radiant heat and light treatment given. Good situation. Every comfort.

### Home for Mental Patient.

WE shall be happy to send on letters to you from any of the Nursing Homes and Doctors' Houses on our Approved List able to receive mental patients, and no doubt you will be able to make a choice from among these.—A. R. C. (155A).

### Masseur Partially Blind.

YOU must be prepared to find it very difficult to get employment. Apply to the National Institution for Massage by the Blind, 188 Marylebone Road, W., but we know they find it hard to place their own pupils. There are no institutions which employ solely blind masseurs. You have a great advantage in being able to see sufficiently to get about without help, and if one of our readers can help you to start, now

### A Neglected Old Woman.

UNHAPPILY you cannot take any action however grievously your poor old friend may be suffering through loneliness and improper feeding. As she will not remain in the workhouse, and as the daughter will not give her proper attention though she lives in the next house, you can only keep watch against actual unkindness, and try the moral effect of unexpected visits. Your district nurse should go in at least twice a week, however coolly received. Send any gifts of food you dispense through the nurse, and this will ensure her a better reception. No, there is no power of detention. The workhouse is not compulsory, and there are grave reasons why it never should be.—DISTRICT VISITOR.

### Hospital Linen League.

IN inviting contributions to the linen store it is quite essential, as you surmise, to specify the prices contributors must pay for the various articles, otherwise the wildest confusion would ensue. Have a list printed or typed out for distribution among the helpers, and state the quantity required, the price, and measurements. As for instance: —100 sheets (linen), 3¼ by 2 yards, price 2s. 3d.; 100 sheets (cotton), 3¼ by 2 yards, price 1s. 6d. to 1s. 9d.; 100 pillow cases (linen), with tapes, not buttons, 35 by 20 inches; 50 blankets (white), 12s. 9d. per pair, etc., etc. For smaller articles it is better to lend patterns. It rather discourages help to prescribe at what shops the articles must be bought. The more free you can leave your helpers the better pleased they will be.—MATRON S. T.

### A Welsh Menu for Convalescents.

THIS is a Convalescent Home for 80 women. Staff, 10. We keep our own cows, a tuberculin-tested herd, and pay 1s. 2d. gallon for milk all the year round; purchase vegetables and fruit grown in our own garden at full market price. Meat, best English (no tenders), veal, pork, beef, and legs mutton, 9d. lb.; lamb, 10d. lb. to 1s. lb.; shoulders and necks mutton, 7d. lb. No fixed diet. I arrange meals with due regard to economy and season of the year, and give great variety. No cold meat or made-up dinners.

*Breakfast.*—Tea or cocoa, bread and butter, with hot ham or bacon, or sausage, or haddock, or cold ham.

*Dinner.*—Fish on Friday, boiled cod or fried plaice, parsley sauce; veal, lamb, pork, beef (roast), beef (boiled), stewed steak, rabbits, mutton (roast), mutton (boiled), mutton (haricot), beef-steak puddings occasionally. Vegetables.—Peas, beans, cabbage, cauliflower, leeks, carrots, turnips, parsnips, onions, vegetable marrow, and potatoes. Sweets.—Fresh fruit tarts, according to season, three times weekly, apple, currant and raspberry, plum, damson, gooseberry, and rhubarb; bread and butter pudding, baked custard tart, cornflour shape and stewed fruit, boiled bread pudding, plum pudding, mince pie, roly-poly jam pudding, raisin suet pudding, semolina, rice, sago, and tapioca pudding.

*Tea.*—Jam, marmalade, celery, lettuce, cake, or potted meat.

*Supper.*—Patients may choose any of the following: Soup, bread and milk, hot milk and bread and butter, porridge, bread and cheese.

In addition to above, patients have one pint milk daily. Total cost per head per day, 10¼d., staff and patients under one heading. The above figure has not varied by more than ¼d. per day during ten years.—"WENDY."

### Discoloured Blankets.

THE difficulty you experience in your laundry through the blankets turning very yellow after steam disinfection is one which is practically insurmountable. A lower temperature than 240° F. would be of no use for disinfection purposes. The only remedy is to bleach the blankets, after disinfection, with sulphur fumes, but in this case be sure to hang them up to bleach while still wet. Perhaps one of our correspondents may be able to suggest another plan.—MANAGERESS.

### Inexpensive Sanatorium.

YOU cannot do better than procure the Memorandum recently issued by the Local Government Board on the construction and arrangement of inexpensive buildings for the reception of consumptive cases. The Board hold to the opinion that no sanatorium can be economically conducted—that is, without serious administrative waste—with fewer than 100 beds. The estimate of £50 per bed for cost of construction furnished in this Memorandum is about half what has often been expended on voluntary institutions for this purpose, but this is for timber framing.—TH. FORBES.

you have obtained your certificates for Swedish massage and medical electricity, we shall be happy to put you in communication. Prepaid letters forwarded to A. C.

### Blind and Deaf.

THE most important thing for you to aim at is to spare your remaining glimmer of sight to the utmost extent. For this reason we have supplied the writing frame, which should enable you, with practice, to correspond readily without looking at the paper. We strongly advise you to master the Braille system, as it will open to you a new world of interest. We have asked a lady to call and help you over this.—J. B.

### Help for a Pensioner.

A GREAT deal of silent distress exists among those who try to live on an insufficient pension. The case you mention might be helped by the Aged Pilgrims' Friend Society, 83 Finsbury Pavement, E.C. It is necessary to subscribe two guineas a year in order to procure a pension, but when a small amount of money can be guaranteed it is wiser to pay it over to this Society, which more than doubles it immediately, and as time goes on often increases the allowance to £7 or £10 a year.—PRACTICAL.

### Mental Nursing in the Transvaal.

YOU cannot practise as a mental nurse in any part of South Africa without obtaining a certificate of competence. The certificate of the Medico-Psychological Association is accepted, but a fee of £1 is charged for registration, and proof of good character, identity, and good health must be produced. It is true the fees are high when employment is obtained, but the work is precarious, and there is much competition. Unless you have good recommendations to doctors in mental practice out there you will not easily make your way.—J. P.

### Institution Facts and Figures.

### Questions for March.

1. GIVE the average cost per head per week for laundry expenses in your institution (*a*) for patients, (*b*) for staff. Mention the number of beds, whether acute medical and surgical cases are received, and whether the washing is done on the premises or by contract outside.

2. Taking any week in the winter months, state what weight of meat was consumed in the institution, less bacon and ham. Divide this amount by the total number of inmates on meat diet, and thus show the average weight of butcher's meat consumed per head per week. In estimating the numbers include both staff and patients, deducting only those on milk or fish, whose diet does not include beef-tea.

The figures must be the result of actual observation and computation, not estimates. Either or both of the questions may be answered by each contributor. The best answers will be published, and for each contribution considered by the Editor to be of sufficient interest for publication payment of FIVE SHILLINGS will be made.

RULES.

The following rules must be observed by all those answering the Housekeeping Questions:—

1. Answers must be written on one side of the paper. They must bear the name and address of the sender and be accompanied by coupon to be cut from the back cover (inside page) of the current issue of THE HOSPITAL. A pseudonym must be chosen if the name is not to be published.

2. Answers must be addressed to the Editor of THE HOSPITAL, 28-29 Southampton Street, Strand, London, W.C.; they must be marked in the left-hand corner "Facts and Figures," and must be received before the end of the month.

### Hints for Convalescent Diet.

WE steam all our potatoes, and always have them mashed and a cup of hot milk mixed with them. When potatoes are not very good they become black on standing, but when mashed are always a good colour, and, I think, are more easily digested. With regard to cooking fish, I always buy cod or ling for patients, and do not have it cut in slices, but have each fish split open the same as a finnan haddock is done, and this is cooked in flat baking tins, just covered with water, and then a few spoonsfuls of dripping are put in with it, and this when baked makes a very appetising dish, the water in which it has been cooked making a kind of gravy, which is always appreciated by children. With regard to typhoid patients, they, when convalescent, have a most liberal diet—three eggs daily, chops, plaice for supper, trifles, boiled and baked custards, chickens, and anything reasonable in season. May I add that I personally superintend all cooking, that I always have plenty of good stock and a liberal supply of dripping? All dripping used for fish is cleansed after use, and used over and over again.—NORTH-COUNTRY FEVER.

### The Editor's Letter-Box.

**Communications have been received and attended to from:—**

Matron A. P.; Home Sister; J. B.; Miss S.; Mrs. St.; H. C. S.; Nurses W. and H., Redhill; Mrs. L. S. Stotfold; Miss F. H. Roydon; A. C.; Miss W.

MRS. T. H. H.—Many thanks for your kind promise of practical service in connection with the case we have commended to you.

APPROVED HOMES AT DERBY AND BRAY.—Please note Rule 3 and enclose communications for the future in separate stamped envelope.

### Editor's Notices.

**Contributions:**

Contributions should be written, or preferably typed, on one side of the paper only, and all articles sent in are accepted upon the distinct understanding that they are forwarded to The Hospital only.

The Editor cannot undertake to return MSS. not used, but when a stamped directed envelope is enclosed the MSS. may be returned if a special request is made.

Accepted articles and paragraphs of news will be paid for after publication at the scale rate.

**Address:**

To prevent delay all contributions and letters on editorial business must be addressed exclusively to the Editor, "The Hospital," 29 Southampton Street, Strand, London, W.C. It is important that this regulation shall be strictly observed.

**Correspondence:**

Correspondence on all subjects is invited. The name and address of correspondents must be given as a guarantee of good faith, but not necessarily for publication.

**Special Articles:**

Special articles are invited, and questions, inquiries, and paragraphs upon all matters relating to the work, administration, and management of general, special, mental, fever, cottage and convalescent hospitals, sanatoria, homes, institutions, societies and organisations for the treatment or care of the sick, injured and dependants of all classes. Every matter affecting the interests and welfare of the staff of all grades working in these institutions will receive special consideration.

**Administrative Medicine, Research and Items of News:**

Special terms will be paid for approved articles on subjects in this wide field by experts who have made some section of it their special study and interest. We wish to give prominence to every movement tending to advance accuracy of diagnosis, efficiency in treatment, and the development of modern methods to eradicate disease and promote the welfare of the suffering.

**A Bureau of Information:**

We invite inquiries and applications for information and help in providing for that numerous class of sufferers who require special care or house-room which they cannot obtain in their own homes or secure for themselves. We cannot, however, prescribe, or recommend practitioners.

**Books for Review:**

Publishers are particularly requested to send advance proofs of any new books of importance whenever possible, as the Editor has made arrangements to publish immediate reviews on a new plan.

**Photographs, Plans, Blocks, and Illustrations:**

It is requested that wherever possible MSS. may be accompanied by illustrations in any of the above forms. The name of the sender and of the article to which it belongs should be written on the back of each photograph, drawing, or block for purposes of identification.

**Local Papers:**

Newspapers containing reports or news paragraphs should be marked and addressed to the Sub-Editor.

**The Coupon System:**

A coupon will be found at the bottom of the third inside page of the cover each week. This coupon must be attached to every question or inquiry to which an answer is desired in The Hospital.

### Lucrative Appointments.

Important and lucrative appointments in all Departments of Institutional Life are announced every week in this section of The Hospital. As its name implies, this Supplement is devoted to the interests of the Institutional Worker, every effort being made to secure information for incorporation in this section of all Vacancies occurring in the Institutional, Poor-Law, and Municipal Services, and the especial attention of our readers is called to the undermentioned appointments, of which full particulars will be found in the following pages:—

Matron (Fever Hospital). Salary commencing at £100 per annum, with uniform, apartments, and board.

Matron (General Hospital). Salary £60 and uniform.

Matron (General Hospital). Salary £90, rising to £100 by annual increments of £5, with board, residence, and laundry.

Matron (Infirmary) (150 beds). Salary £100 per annum, advancing by increments of £5 annually to a maximum of £120.

Matron (Counties Asylum). Salary £70 per annum, rising by £5 annually to £100, with apartments, board, laundry, and an allowance for uniform.

Charge Nurse (Infirmary). Salary at rate of £30 per annum, with board, residence in Nurses' Home, uniform, and laundry.

Staff Nurse (Out-patient Department). Salary £30 and uniform.

Nurse (Infirmary). Salary £30, with indoor uniform.

If you cannot find among the announcements a post suited to your experience and capabilities, there is the Appointments Wanted Column, which will enable you to bring your requirements directly to the notice of the Governing Bodies and Controlling Officials of Institutions of every description throughout the country for the small fee of 1s. for 21 words.

### Manager's Notices.

**Letters relating to the publication, sale, and advertising departments of "The Hospital" must be addressed to The Manager, "The Hospital," The Hospital Building, 28 and 29 Southampton Street, London, W.C.**

**Subscriptions** may begin at any time, and are payable in advance. Cheques and Post-Office Orders should be crossed London County and Westminster Bank, Covent Garden Branch, and made payable to the Manager of The Hospital.

Rates of Subscription (including Postage).

| *United Kingdom.* | | *Foreign and Colonial.* | |
|---|---|---|---|
| Three Months ... | ... 2s. 0d. | Three Months ... | ... 2s. 6d. |
| Six Months ... | ... 4s. 0d. | Six Months ... | ... 5s. 0d. |
| Twelve Months ... | ... 6s. 6d. | Twelve Months ... | ... 8s. 8d. |

**Special Rates** will be quoted for those institutions that agree to send all their vacancy, contract, and public notices to The Hospital each year, so as to make it a file of such announcements for ready reference.

*SUBSCRIPTION FORM.*

*Please send me* The Hospital *for..............months, commencing with your issue of............................, for which please find enclosed............................*

*Name*..........................................................

*Address* ......................................................

......................................................

*Date*..........................................

*To* ........................................ *Newsagent.*

**Remittances:**

**It is especially requested that remittances be made by Cheque or Postal Order, and in halfpenny stamps only when the amount is under sixpence.**

